# Human Cathelicidin Production by the Cervix

**DOI:** 10.1371/journal.pone.0103434

**Published:** 2014-08-04

**Authors:** Lorraine Frew, Sofia Makieva, Andrew T. M. McKinlay, Brian J. McHugh, Ann Doust, Jane E. Norman, Donald J. Davidson, Sarah J. Stock

**Affiliations:** 1 Tommy's centre for Maternal and Fetal Health, MRC Centre for Reproductive Health, The University of Edinburgh, Edinburgh, United Kingdom; 2 MRC Centre for Inflammation Research, The University of Edinburgh, Edinburgh, United Kingdom; Charité-University Medicine Berlin, Germany

## Abstract

hCAP18/LL-37 is the sole human cathelicidin; a family of host defence peptides with key roles in innate host defence. hCAP18/LL-37 is expressed primarily by neutrophils and epithelial cells, but its production and function in the lower genital tract is largely uncharacterised. Despite the significant roles for cathelicidin in multiple organs and inflammatory processes, its impact on infections that could compromise fertility and pregnancy is unknown. The aim of this study was to investigate cathelicidin production, regulation and function in the cervix. hCAP18/LL-37 was found to be present in cervicovaginal secretions collected from women in the first trimester of pregnancy and to be expressed at significantly higher levels in samples from women with alterations in vaginal bacterial flora characteristic of bacterial vaginosis. In endocervical epithelial cell lines, expression of the gene encoding hCAP18/LL-37 (*CAMP*) was not affected by TLR agonists, but was found to be up-regulated by both 1, 25 hydroxyvitamin D_3_ and 25 hydroxyvitamin D_3_. However, no association was found between serum levels of vitamin D and hCAP18/LL-37 concentrations in cervicovaginal secretions (n = 116). Exposure to synthetic LL-37 had a pro-inflammatory effect on endocervical epithelial cell lines, increasing secretion of inflammatory cytokine IL-8. Together these data demonstrate inducible expression of hCAP18/LL-37 in the female lower reproductive tract *in vivo* and suggest the capacity for this peptide to modulate host defence to infection in this system. Further investigation will elucidate the effects of hCAP18/LL-37 on the physiology and pathophysiology of labour, and may lead to strategies for the prevention of infection-associated preterm birth.

## Introduction

Cathelicidins are an evolutionary conserved family of multifunctional host defence peptides (HDP) [1]. hCAP18/LL-37 is the sole human cathelicidin and has important roles in innate host defence [2]. Predominantly expressed by neutrophils and epithelial cells, hCAP18/LL-37 expression can be induced by bacterial products and inflammatory stimuli [3], [4], and can be regulated by vitamin D_3_ [5], [6].

LL-37 has been shown to be a component of cervicovaginal secretions [7]–[9], but little is known about its production and function in pregnancy. The lower genital tract is continually exposed to both the external environment and to pathogenic bacteria, yet infections are relatively rare in this environment. Epithelial cells of the vagina and cervix create a physical barrier to infection, and the normal vaginal population of lactobacilli create an inhospitable environment for invading pathogens [10]. Altered expression of other HDP and antimicrobial proteins in pregnancy has been associated with both bacterial vaginosis in pregnancy [11], [12], a condition characterized by abnormal vaginal flora, and with preterm labour. Recently HDP have been shown to be critical to the ability of the cervix to prevent ascending infection [13].

We hypothesise that hCAP18/LL-37 is produced by cervical epithelial cells, and that altered expression levels of LL-37 may be associated with infection and inflammation in the female lower reproductive tract. The aim of this study was to investigate the production and function of hCAP18/LL-37 expressed by female lower genital tract epithelium using *in vivo* and *in vitro* techniques.

## Materials and Methods

### Ethical approval and consent

Ethics approval was obtained from Lothian Local Research Ethics (REC: Reference number 04/S1101/24) and Edinburgh Reproductive Tissue Biobank (REC: Reference number 09/S0704/3). Informed written consent was obtained from participants who donated samples.

### Sample collection

Matched cervicovaginal secretions (CVS) and serum samples were obtained from the Edinburgh Reproductive Tissue Biobank. These samples had been collected from women when they attended for their first trimester screen for Down's syndrome at the Simpson's Centre for Reproductive Health at the New Royal Infirmary of Edinburgh. Inclusion criteria for this study were singleton pregnancy at 11–14 weeks gestation. Exclusion criteria were factors that may affect LL-37 levels in cervicovaginal secretions; current or recent urinary tract or sexually transmitted infection, antibiotic use within the previous two weeks, sexual intercourse within 48 hours and current smoker status. Samples were self-collected by cotton swab. Women were instructed to rotate the swab in the vagina for 10 seconds then remove to 750 µl buffer solution (protease inhibitor cocktail tablet (Roche Diagnostics, Indianapolis, USA) in 10 mls Phosphate Buffered Saline (PBS; Gibco Life Technologies Ltd, Paisley, UK). Samples were centrifuged at 2000 g×3 min, 2000 g×5 min, and supernatant was passed through a 0.2 µM pore syringe filter and stored at −80°C. A second swab was used to make a vaginal smear on a glass slide and allowed to air dry, for subsequent Gram stain (BIOS Europe, Skelmersdale, UK). Diagnosis of normal flora, intermediate flora or bacterial vaginosis was made using Nugent's criteria [14].

Peripheral venous blood was drawn from an antecubital vein with a 21-guage needle into a Sarstedt Monovette serum-gel blood collection tube (Sarstedt, Numbrecht, Germany) for the isolation of serum. Samples were stored on ice and processed within 20 min of collection. Samples were centrifuged at 1500 g at 4°C for 15 minutes. Serum was drawn off and centrifuged at 1500 g at 4°C for 15 minutes before being stored at −80°C.

### ELISA

The amount of total protein in cervicovaginal secretion samples was measured using a commercial assay (Bio-Rad Laboratories, Hemel Hempstead, UK) according to the manufacturer's protocol. Myeloperoxidase (MPO; range 0.4 ng/ml to 100 ng/ml, inter-assay precision 4.4%) and hCAP18/LL-37 (range 0.1 ng/ml to 100 ng/ml, inter-assay precision 12.8%) assays were performed with ELISA kits from Hycult (HycultBiotechnology, The Netherlands) and 25 hydroxyvitamin D enzyme immuno assay (range 6.7 nmol/L to 300 nmol/L, inter-assay precision 12.8%) was performed with a kit from Immunodiagnostics (Immunodiagnostics Systems Ltd, Bolden, UK). IL-8 (range 31.3 pg/ml to 2000 pg/ml, inter-assay precision 14.7%) assays were performed using matched antibody pairs from R&D (R&D systems, Oxford, UK) according to manufacturers protocols.

### Western blot

Western blotting was used to detect hCAP18/LL-37 protein in cervicovaginal secretions. Protein was separated by SDS-PAGE and was then transferred to polyvinylidene difluoride (PVDF) membranes. Membranes were blocked for 1 h at room temperature with 5% dry skimmed milk in 50 mM Tris-HCl, pH 7.5, 150 mM NaCL, 0.2% Tween-20, and were then probed overnight at 4°C with rabbit polyclonal anti-LL-37 (1∶1000) (PA-LL-37-100, Innovagen, Lund, Sweden) followed by extensive washing. Immunoreactive materials were detected by enhanced chemiluminescence using HRP conjugated anti-rabbit secondary antibody (1∶1000). Membranes were incubated with chemiluminescent developing solution (ECL Western Blot Analysis System, GE Healthcare, Buckinghamshire, UK) and exposed to X-ray film for 30 seconds, before the film was exposed using an X-ray developer.

### Cell culture

END E6/E7 and ECT E6/E7 (LGC Promochem, Teddington, UK) were cultured in keratinocyte serum-free media (Invitrogen, Paisley, UK), supplemented with 0.1 ng/ml human recombinant epidermal growth factor (Invitrogen), 0.05 mg/ml bovine pituitary extract (Invitrogen) and 0.4 mmol/l calcium chloride (Sigma-Aldrich). They were plated at 0.2×10^6^ cells/ml in 1 ml in 12-well plates and maintained at 37°C at 95% air, 5% CO_2_. Cells were treated with Pam3CSK4 (InvivoGen, San Diego, CA), POLY (I:C) (InvivoGen), recombinant flagelin (Rec FLA-ST) (InvivoGen), FSL-1 (InvivoGen), 1, 25 dihydroxyvitamin D_3_ (Sigma-Aldrich), 25 hydroxyvitamin D_3_ (Sigma-Aldrich), synthetic LL-37, D LL-37 (the D enantiomer) or scrambled LL-37 (RSLEGTDRFPFVRLKNSRKLEFKDIKGIKREQFVKIL; all custom synthesised by Almac [East Lothian, Scotland] using Fmoc solid phase synthesis and reversed phase HPLC purification, with identity confirmed by electrospray mass spectrometry, purity [>95% area] by RP-HPLC and net peptide content determined by amino acid analysis), lipopolysaccharide (LPS) (Sigma-Aldrich), recombinant human IL-1β (Peprotech EC), pertussis toxin (PTX) (Calbiochem, Darmstadt, Germany), WRW4 (Calbiochem), KN-62 (Sigma-Aldrich) and oxidised ATP (oATP) (Sigma-Aldrich). Treatments were performed in duplicate. RNA was extracted for analysis by TaqMan quantitative polymerase chain reaction (PCR). Media was harvested and analysed by ELISA, as described above.

### RNA extraction and quantitative real-time (qRT)-PCR

RNA was extracted using the RNeasy mini system (Qiagen, Crawley, UK) according to manufacturer's protocols. RNA concentration and purity were assessed using the Nanodrop ND-1000 spectrophotometer (Thermo Scientific), with a 260∶280 absorbance value between 1.9 and 2.1 considered satisfactory. RNA samples were reverse transcribed with the use of random primers (High capacity cDNA reverse transcription kit; Applied Biosystems, Life Technologies Ltd, Paisley, UK), and amplified by ABI Prism TaqMan 7900 (Applied Biosystems), according to standard protocol. Target mRNA was quantified in relation to 18S ribosomal RNA abundance in each sample. Negative controls were: i) a negative reverse transcribed-sample (RNA template with no reverse transcriptase enzyme); ii) reverse transcribed-H2O (water in place of RNA template); and iii) a TaqMan-reaction negative control (complementary DNA replaced with H2O). Expression of *CAMP* and *DEFB4* were measured using pre-designed TaqMan assays (Applied Biosystems).

### Statistical analysis

A sample size calculation was performed based on standard deviation of a similar HDP (HBD-2) being 2.56 ng/ml (Stock, unpublished) and the standard deviation of vitamin D in Caucasian pregnant women of 8.5 ng/ml [15]. A sample size of 117 patients had 80% power for a minimal detectable difference of 800 pg in LL-37 concentrations, at 0.05 significance. 800 pg was chosen as the minimal detectable difference of interest, based on a study which found a reduction in epithelial defensins of approximately this magnitude was correlated with a decrease in antibacterial activity [16].

Statistical analysis was carried out using the program Graphpad Prism (GraphPad Software Inc, San Diego, CA, USA). Relationships between cervicovaginal hCAP18/LL-37, myeloperoxidase and serum 25(OH) vitamin D were analysed with Pearson's correlation. Quantitative RT-PCR data was analysed with one-way ANOVA. ELISA data was analysed with 2-way ANOVA. Differences were considered statistically significant at P<0.05.

## Results

### Sample cohort

From 199 samples of cervicovaginal secretions available in the biobank 130 were eligible for inclusion. The mean maternal age was 39 years (standard deviation 5.2) and the mean gestational age sampling was 13 weeks (standard deviation 8 days). 126/130 (95%) of the cohort were white. The median Scottish Index of maternal deprivation category (based on postcodes) was 4 (range 3–5).

### hCAP18/LL-37 in cervicovaginal secretions

**Figure 1:**
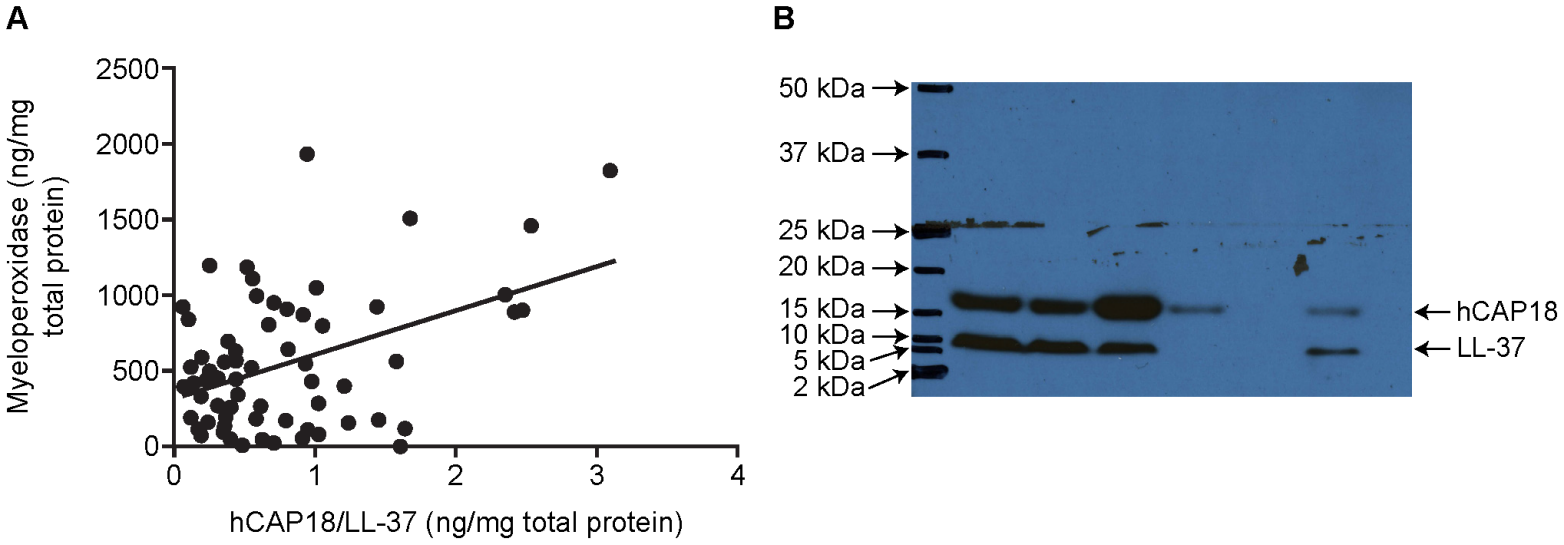
Cervical hCAP18/LL-37 expression. (**A**) Correlation between cervicovaginal hCAP18/LL-37 and Myeloperoxidase (r = 0.1; n = 77; p = 0.3). (**B**) Representative western blot of hCAP18/LL-37 expression in cervicovaginal secretions. The 18 kDa hCAP18 and the 5–10 kDa cleaved peptide LL-37 were detected in cervicovaginal secretions.

hCAP18/LL-37 was detected in 79/130 (60.8%) samples of cervicovaginal secretions with a median concentration of 0.3 ng/mg total protein (range 0.14–0.68 ng/mg total protein). To investigate whether neutrophils were the main source of hCAP18/LL-37 we examined the correlation between myeloperoxidase (MPO), a neutrophil granule product, and hCAP18/LL-37. A weak positive correlation was found between cervicovaginal MPO and cervicovaginal hCAP18/LL-37 (r = 0.1; n = 77, p = 0.3), the limited strength suggesting that neutrophils were not the predominant source of hCAP18/LL-37 (Figure 1A).

As the hCAP18/LL-37 ELISA does not distinguish between the pro-peptide hCAP18 and the cleaved peptide LL-37, western blotting was performed on a subset of 48 samples. Bands were identified at 18 kDa, representing the pro-protein hCAP18, and between 5 and 10 kDA, representing cleaved LL-37. Samples were found to have both pro-peptide and cleaved LL-37, pro-peptide only, or neither. Figure 1B shows a representative gel with 7 individual cervicovaginal secretion samples.

### hCAP18/LL-37 and bacterial vaginosis

In 116 cases slides were suitable for analysis of bacterial flora using Nugent's criteria for diagnosis of Bacterial Vaginosis. 74/116 (63.8%) of women had normal flora, 17/116 (14.6%) had intermediate flora and 25/116 (21.6%) had bacterial vaginosis. There were no significant differences in mean age, ethnicity, gestation at sampling or deprivation category between the groups.

**Figure 2:**
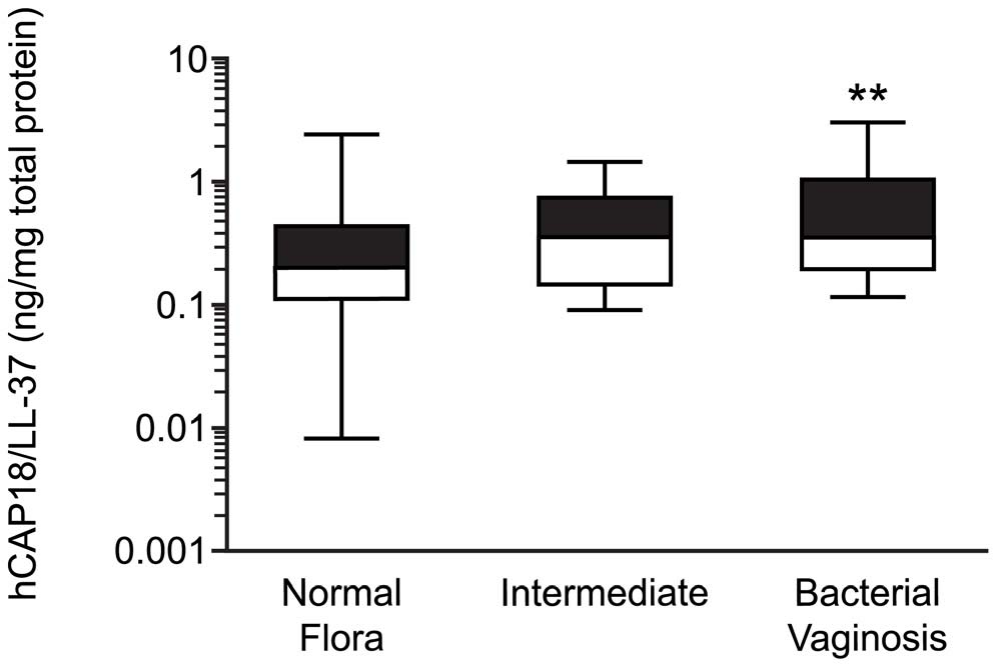
hCAP18/LL-37 expression in cervicovaginal secretions of women with and without Bacterial Vaginosis. hCAP18/LL-37 concentration in women with normal flora, intermediate vaginal flora and bacterial vaginosis. Data presented as median ± interquartile range. **, p<0.01 (One-way ANOVA with Dunnett's post-test) compared to those with normal flora.

Total hCAP18/LL-37 was significantly higher in samples from women with bacterial vaginosis compared to women with normal flora (median concentration 0.35 ng/mg total protein; interquartile range 0.2–1 ng/mg total protein, compared to 0.2 ng/mg total protein interquartile range 0.12–0.45 ng/mg total protein, p<0.01) (Figure 2). 37/74 (50%) of women with normal flora, 7/17 (41.2%) of women with intermediate flora and 8/25 (32%) of women with bacterial vaginosis had undetectable levels of hCAP18/LL-37. There was no correlation between the presence of bacterial vaginosis and the cleavage forms of cathelicidin peptide identified on western blotting (hCAP18 pro-peptide or LL-37 cleaved peptide) (data not shown).

### Regulation of LL-37 production by epithelial cells of the lower genital tract

Having shown that hCAP18/LL-37 is a component of cervicovaginal secretions, and that levels are associated with changes in bacterial flora, we examined expression of the *CAMP* gene (encoding for hCAP18/LL-37) *in vitro*, using cell lines derived from the endocervix (END E6/E7) and ectocervix (ECT E6/E7). Transcription of *DEFB4* (encoding the related peptide HBD-2) was included as a comparison.

**Figure 3:**
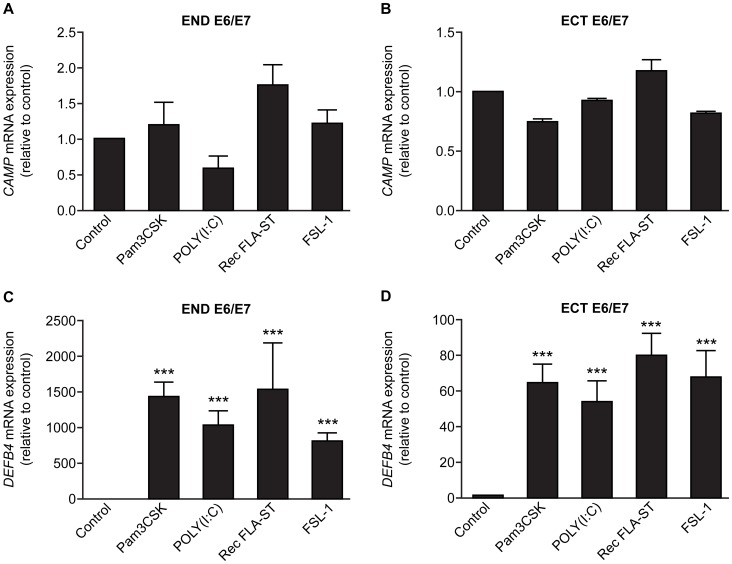
Effect of TLR agonists on *CAMP* and *DEFB4* expression in endocervical (END E6/E7) and ectocervical (ECT E6/E7) cell lines. Cells cultured for 24(250 ng/ml), POLY (I:C) (5 ng/ml), Rec FLA-ST (100 ng/ml) and FSL-1 (100 ng/ml), and untreated controls. (A) END E6/E7 *CAMP* expression (n = 3), (B) ECT E6/E7 *CAMP* expression (n = 3), (C) END E6/E7 *DEFB4* expression (n = 3), (D) ECT E6/E7 *DEFB4* expression (n = 3). Data presented as mean fold change relative to control ± SEM. **, ***, P<0.01, 0.001 respectively compared with control. (One-way ANOVA with Dunnett's post-test).

**Figure 4:**
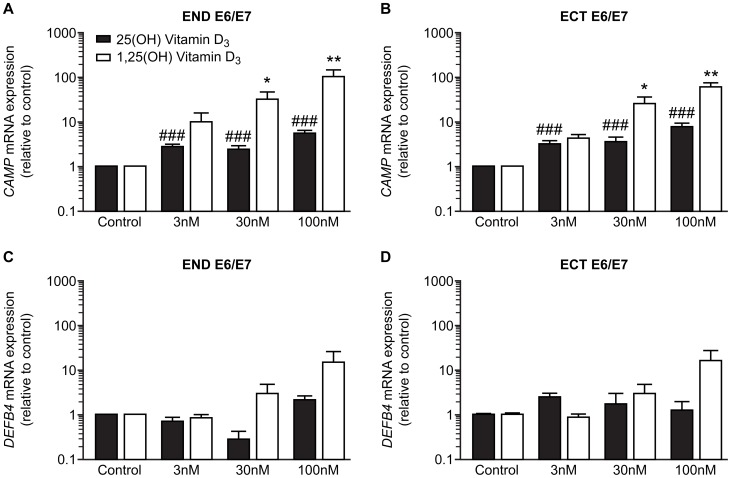
Effect of vitamin D on *CAMP* and *DEFB4* expression in endocervical (END E6/E7) and ectocervical (ECT E6/E7) cell lines. Cells treated for 24(control), 3 nM, 30 nM or 100 nM of 25(OH) vitamin D_3_ or 1,25 (OH) vitamin D_3_. (A) END E6/E7 *CAMP* expression (n = 3), (B) ECT E6/E7 *CAMP* expression (n = 3), (C) END E6/E7 *DEFB4* expression (n = 3), (D) ECT E6/E7 *DEFB4* expression (n = 3). Data presented as mean fold change relative to control ± SEM. ##, ###, p<0.01, 0.001 respectively compared to 25 (OH) vitamin D_3_ control. *, **, p<0.05, 0.01 compared to 1,25(OH) vitamin D control_3_. (One-way ANOVA with Dunnett's post-test).

**Figure 5:**
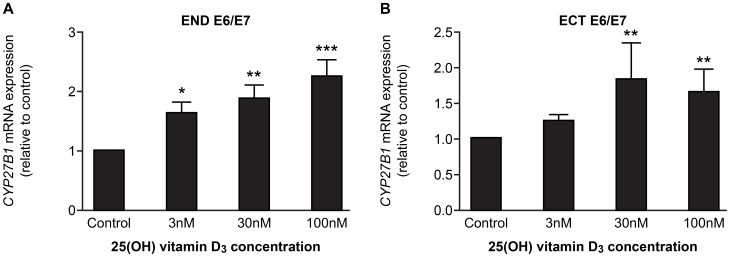
Effect of 25(OH) vitamin D_3_ on *CYP27B1* expression in endocervical (END E6/E7) and ectocervical (ECT E6/E7) cell lines. Cells treated for 24(control), 3 nM, 30 nM or 100 nM 25(OH) vitamin D_3_. (A) End E6/E7 cells (n = 3), (B) Ect E6/E7 cells (n = 3). Data presented as mean fold change relative to control ± SEM (error bars). *, **, **, p<0.05, 0.01, 0.001 respectively compared with control (One-way ANOVA with Dunnett's post-test).

Treatment with TLR agonists; Pam3CSK (TLR 1/2 agonist), POLY (I:C) (TLR 3 agonist), Rec FLA-ST (TLR 5 agonist) and FSL-1 (TLR 2/6 agonist), had no effect on *CAMP* expression in END E6/E7 and ECT E6/E7 cells compared to untreated controls, but markedly up-regulated expression of *DEFB4* (Figure 3). In contrast, treatment with 25(OH) vitamin D_3_ and 1, 25(OH) vitamin D_3_ up-regulated the expression of *CAMP*, but not *DEFB4* in END E6/E7 and ECT E6/E7 cell lines (Figure 4). Treatment with 25(OH) vitamin D_3_ also increased the expression of the gene encoding 25-hydroxyvitamin D-1α-hydroxylase (*CYP27B1*) which catalyses the conversion of 25(OH) vitamin D_3_ to 1, 25(OH) vitamin D_3_ in each cell line (Figure 5).

### Serum vitamin D levels and hCAP18/LL-37 concentrations in cervicovaginal secretions

Given the observation that vitamin D_3_ influenced *CAMP* gene expression *in vitro*, we sought to determine whether serum vitamin D concentration was associated with hCAP18/LL-37 levels in cervicovaginal secretions. Matched serum samples were available for 122 women from our original cohort for vitamin D analysis.

**Figure 6:**
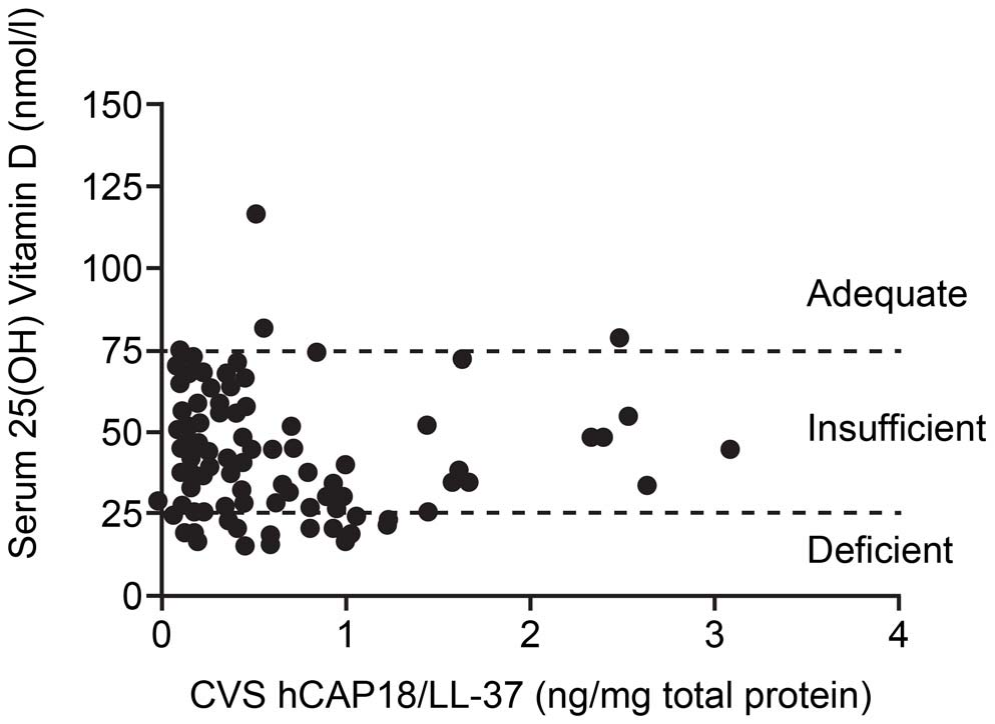
Relationship between 25(OH) vitamin D and hCAP18/LL37 in endocervical (END E6/E7) and ectocervical (ECT E6/E7) cell lines. Correlation between serum 25(OH) vitamin D and CVS hCAP18/LL-37 (r = −0.13; n = 122; p = 0.14). Data presented as scatter plots and divided in to deficient <25 nmol/l, insufficient 25–75 nmol/l and adequate >75 nmol/l. Data analysed by Pearson's Correlation.

Serum 25(OH) vitamin D levels were measured and categorised into three groups; deficient (25 nmol/l), insufficient (25–75 nmol/l) and adequate (.75 nmol/l) as previously described [17]. Overall, 13/122 (10.6%) of sample women were deficient, 105/122 (86.1%) insufficient and 4/122 (3.3%) had adequate 25(OH) vitamin D levels. The mean age of women participating in this study was 32 years and this was not different between women deficient, insufficient and adequate for serum 25(OH) vitamin D. 57/122 (46.7%) women were taking vitamin supplements at the time of sample collection, those taking supplements had significantly higher levels of serum 25(OH) vitamin D compared to those not taking supplements (data not shown, mean concentration 38.6 nmol/l ± SEM 1.9, compared to 17 nmol/l ± SEM 2.3, p<0.001). Samples were collected from women from July 2011 until March 2012 and when analysed the time of year of sample collection did not affect whether women had deficient, insufficient or adequate levels of circulating 25 (OH) vitamin D. No correlation between serum 25(OH) vitamin D and cervicovaginal hCAP18/LL-37 concentration was detected (r = −0.13; n = 122; p = 0.14) (Figure 6).

### The effect of LL-37 on cytokine expression released by end cell line

Finally, we investigated whether LL-37 peptide had any effect on the inflammatory response of cells derived from the cervical epithelium. Initial experiments demonstrated that LL-37 exposure increased production of IL-8 and IL-6 by END E6/E7 cells in a concentration-dependent manner with an EC_50_ of 23.9 µg/ml and 20.2 µg/ml respectively (Figure S1). For all subsequent experiments a concentration of 25 µg/ml LL-37 was used.

Treatment of END E6/E7 cells with 25 µg/ml LL-37 resulted in an increase in IL -8 at 4, 6 and 24 hours (P<0.001) and IL-6 at 6 and 24 hours (P<0.05, P<0.001 respectively) compared to untreated control (Figure S2). The effect of LPS (1 µg/ml) and IL-1β (10 ng/ml) were included as positive controls, and the response to 25 µg/ml LL-37 was found to be of similar or greater magnitude (Figure S2).

To determine specificity of the IL-8 response to LL-37, we cultured END E6/E7 and ECT E6/E7 cells for 24 hours with LL-37 or scrambled LL-37. Cells treated with scrambled LL-37 (containing the same amino acids as LL-37 rearranged in a scrambled order), had no effect on IL-8 secretion in END E6/E7 or ECT E6/E7 cells (Figure S3). Similarly cells treated with a D LL-37 enantiomer of LL-37 had no effect on IL-8 secretion (Figure S3), suggesting this effect of LL-37 is likely to be specific and receptor mediated.

**Figure 7:**
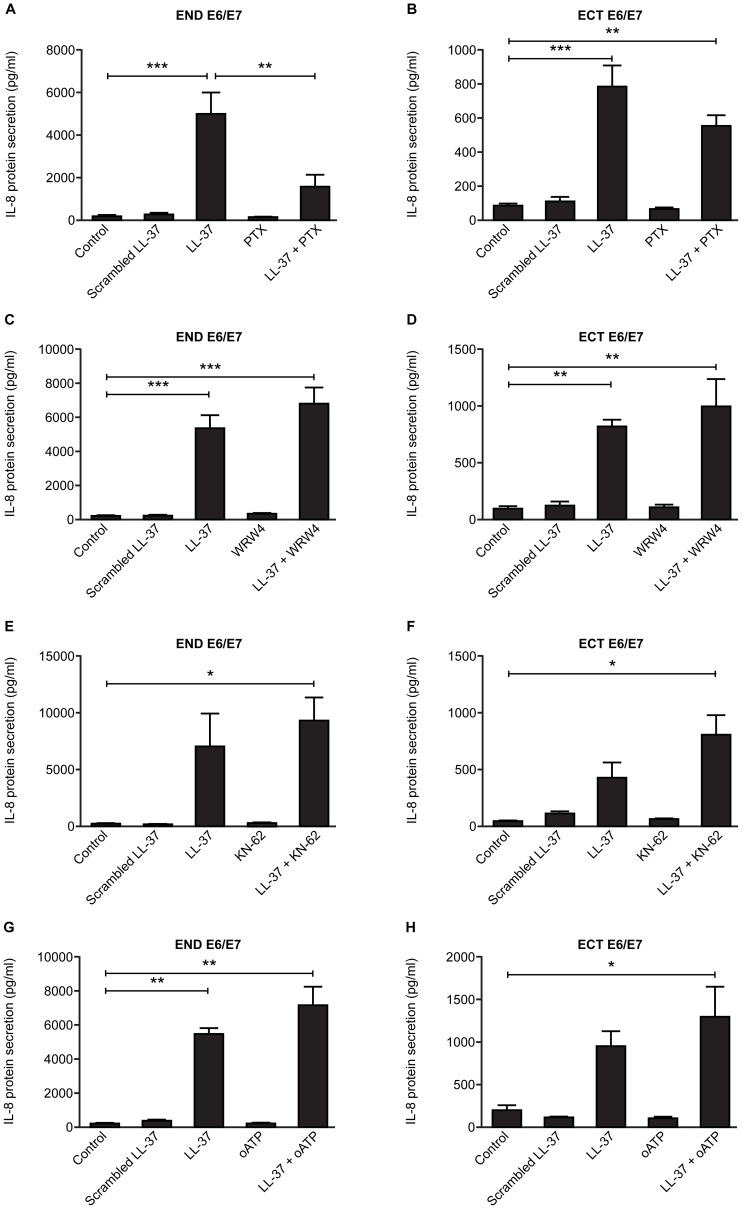
Effect of LL-37 receptor antagonists on IL-8 secretion in endocervical (END E6/E7) and ectocervical (ECT E6/E7) cell lines. END E6/E7 cells were pre-treated for 30 minutes with or without antagonists before being cultured for 24 hours with scrambled LL-37 (25 µg/ml) and LL-37 (25 µg/ml). (A) END E6/E7 treated with PTX (GPCR antagonist, 200 ng/ml), (B) ECT E6/E7 cells treated with PTC, (C) END E6/E7 cells treated with WRW4 (FPR2 antagonist, 10 µM), (D) ECT E6/E7 cells treated with WRW4, (E) END E6/E7 cells treated with KN-62 (P2X_7_R antagonist, 10 µM), (F) ECT E6/E7 cells treated with KN-62, (G) END E6/E7 cells treated with oATP (P2X_7_R antagonist, 100 µM) and (H) ECT E6/E7 cells treated with oATP. Data presented as mean concentration ± SEM (error bars). *, **, ***, p<0.05, 0.01, 0.001 respectively. (2way ANOVA with multiple comparison tests).

Antagonists to specific receptors proposed to mediate some of the effects of LL-37 were used to investigate the mechanisms involved in these LL-37 mediated cytokine responses. Co-treatment with the G-protein coupled receptor (GPCR) antagonist pertussis toxin (PTX) resulted in lower levels of LL-37 induced IL-8 secretion by END E6/E7 cells compared to LL-37 treatment alone (P<0.01, Figure 7A). There was no difference in IL-8 secretion in ECT E6/E7 cells treated with PTX and LL-37 compared to LL-37 alone (Figure 7B). Treatment with specific antagonists to formyl peptide receptor (FPR2) and the purinergic P2X_7_ receptor had no effect on LL-37 induced IL-8 expression in END E6/E7 and ECT E6/E7 cells (Figure 7C-H).

## Discussion

In this study we have shown for the first time that total hCAP18/LL-37 concentration is increased in cervicovaginal secretions from women with bacterial vaginosis. The increase of hCAP18/LL-37 may be in response to bacterial flora changes, in order to protect the genital tract either by direct killing of the bacteria, or through modulation of innate immune defences. An alternative hypothesis is that high levels of LL-37 could predispose to bacterial vaginosis. However, other studies of antimicrobial polypeptides have shown normalisation in levels after treatment of bacterial vaginosis [16] suggesting that alterations in levels occur as a protective measure in response to bacterial vaginosis, rather than being causative of it.

The lack of association between hCAP18/LL-37 and MPO levels, led us to conclude that neutrophils were not the main source of the peptide in the lower genital tract and to study expression by epithelial cells. Despite evidence of an association with bacterial vaginosis, TLR agonists did not regulate LL-37 gene expression in cells derived from epithelia of the lower genital tract. This is in contrast to other studies which have shown that expression of *CAMP* is increased in response to TLR agonists, including POLY (I:C) and peptidoglycan, in other epithelial cell lines [18], [19]. This suggests different *CAMP* responses depending on cell type and environment. In contrast, TLR agonists did regulate expression of the related host defence peptide, HBD-2, and TLRs are expressed by these cells (data not shown). One explanation for this could be that the lack of normal microflora (lactobacilli) that occurs in bacterial vaginosis may stimulate LL-37 levels, rather than presence of other bacteria. Alternatively, it has been suggested that TLR-mediated up-regulation of *CAMP* could be dependent upon the presence of vitamin D. In human macrophages it has been demonstrated that TLR stimulation induces expression of CYP27B1, the vitamin D receptor and the expression of *CAMP* [20]. TLR signalling leading to stimulation of vitamin D expression has also been shown to synergistically induce *CAMP* expression in human neutrophils, macrophages and monocytes [5], [21], [22]. Investigating the expression of *CAMP* in response to co-stimulation with TLR agonists and vitamin D in END E6/E7 and ECT E6/E7cells, we found no change in the induced expression of *CAMP* (data not shown).

*CAMP* expression was increased by exposure to vitamin D_3_, and END E6/E7 and ECT E6/E7 cell lines expressed the enzyme CYP27B1, which converts inactive 25 (OH) vitamin D_3_ to active 1, 25 (OH) vitamin D_3_. Stimulation of *CAMP* expression by both 25(OH) vitamin D_3_ and 1, 25(OH) vitamin D_3_ suggests that these cells can convert vitamin D_3_ to its active form in an autocrine manner, where treatment with 25(OH) vitamin D_3_ stimulates the production of CYP27B1, essential in the production of 1,25 (OH) vitamin D_3_. Increased *CAMP* expression in response to vitamin D_3_ has been shown in other cell types including acute myeloid leukaemia, immortalized keratinocyte, colon cancer cell lines, bone marrow derived macrophages [6] and airway epithelial cells [23], [24]. These data raise the possibility that vitamin D sufficiency *in vivo* might be an important factor affecting the capacity to up-regulate hCAP18/LL-37 expression in the reproductive tract, with implication for responsiveness/susceptibility to infection.

The *in vivo* data from out study did not show a relationship between serum 25(OH) vitamin D and circulating or cervicovaginal hCAP18/LL-37 levels in pregnant women at 11-14 weeks gestation. However, there were only four women who had adequate levels of vitamin D in our cohort (based on a report by [17]), despite advice that all pregnant women in Scotland take vitamin D supplementation [25]. This clearly places a limitation on our ability to draw conclusions about the effects of vitamin D on hCAP18/LL-37 expression, and any consequent effects on bacterial vaginosis in our cohort. Samples were collected from patients from July 2011 until March 2012, when analysed the time of year of sample collection did not affect whether women had deficient, insufficient or adequate levels of circulating 25(OH) vitamin D (data not shown). It has been well documented that the critical importance of latitude and climate on sunlight exposure on vitamin D synthesis has led to concern in Scotland over the vitamin D status of the general population and pregnant women in particular [26]. A recent study conducted in pregnant women in Dublin, a similarly northerly latitude to Scotland, found suboptimal vitamin D status to be common in a cohort of pregnant women [27].

Other studies reporting the relationship between serum 25(OH) vitamin D and hCAP18/LL-37 concentration have generated conflicting results. A study conducted by Dixon et al. in healthy adults identified a positive correlation between circulating plasma hCAP18/LL-37 and serum 25(OH) vitamin D in subjects with 25(OH) vitamin D levels ≤80 nmol/L as opposed to those with concentrations >80 nmol/L [28]. Bhan et al. also found a positive correlation between serum 25(OH) vitamin D levels <80 nmol/l and baseline hCAP18/LL-37 levels, and changes in hCAP18/LL-37 levels after high-dose vitamin D_2_, ergocalciferol treatment [29]. In the pregnant cohort we studied, 121 out of 122 women had serum 25(OH) vitamin D levels <80 nmol/l, therefore we could not examine this relationship. A positive correlation between vitamin D levels and hCAP18/LL-37 has also been observed in a study of critically ill patients with and without sepsis [30]. In contrast, studies conducted in dialysis patients [31] and patients with bone disease [32] did not observe a correlation between serum 25(OH) vitamin D and circulating hCAP18/LL-37 levels, reflecting the observations obtained from our pregnant cohort. This is further supported by a study in maternal plasma obtained prior to caesarean section or shortly after vaginal delivery at term, where no relationship was discovered between 25(OH) vitamin D and plasma hCAP18/LL-37 concentration in either of these delivery groups, nor was a relationship found in infant blood samples from umbilical cords collected following delivery [33].

A study of cathelicidin expression in the urinary tract found that supplementation with 25(OH) vitamin D for 3 months did not up-regulate *CAMP* expression in serum or in bladder tissue obtained from participants, but the ability to produce *CAMP* in response to uropathogenic *E.coli* was altered following vitamin D supplementation [34]. Although we did not observe a correlation between the concentration of cathelicidin and circulating 25(OH) vitamin D in this study, it would be interesting to investigate the expression of cathelicidin from cervicovaginal secretions following exposure to genital tract pathogens, and assess if *CAMP* expression is altered in women with deficient, insufficient and adequate vitamin D levels. The effect of vitamin D on LL-37 function in the lower genital tract was outwith the scope of this study, but would be an interesting area of future research.

We found that LL-37 has a pro-inflammatory effect on epithelial cells derived from the endocervix, producing IL-8 and IL-6 in response to LL-37. The concentration of LL-37 used in our *in vitro* study is typical of other related studies in the literature and within the physiological range proposed for other systems, including the lung [35]. Due to the nature of cervicovaginal sample collection by vaginal swab and LL-37 being a cationic peptide which is thought to closely adhere to epithelial surfaces due to it's negative charge, it is difficult to extrapolate from cervicovaginal samples to the physiological levels present at cervical epithelial surfaces.

Two receptors that have been proposed to be involved in mediating the effects of LL-37 in various cells are FPR2 and a P2X_7_ receptor [36]–[38]. The only receptor thought to bind LL-37 in a classical receptor-ligand mechanism is the Gi protein coupled receptor, FPR2. This interaction was first identified as a mechanism for LL-37-mediated chemotaxis of leukocytes [36], [39], but has also been implicated in a number of LL-37-mediated roles including wound healing [40], angiogenesis [41], modulation of neutrophil death [42], [43] and in activating MAPK and enhancing invasiveness of ovarian carcinoma cells [44]. P2X_7_R has been implicated as a receptor for LL-37, where LL-37 treatment of LPS primed monocytes leads to post-translational modification and release of IL-1β via P2X_7_R in a dose dependent manner [37], [45]. This receptor has also been shown to be involved in LL-37-mediated modulated neutrophil death [43], [46], endothelial cell stiffening [47], wound healing [48] and IL-8 production in human gingival fibroblasts [49]. When cells derived from the endocervix were treated with PTX, a GPCR inhibitor, LL-37 mediated induction of IL-8 production was inhibited, however the specific FPR2 inhibitor, WRW4, and the P2X_7_R antagonists, oATP and KN-62, did not inhibit the LL-37-mediated increase in IL-8, indicating that this effect is mediated through an un-identified GPCR. Other LL-37 effects have been shown to be mediated through as-yet-unidentified GPCRs [50], where they are implicated in LL-37 mediated modulation of dendritic cell differentiation [51], also based on inhibition of LL-37 mediated effects by PTX. Another alternative receptor is GAPDH, that has been identified as a novel intracellular receptor, shown to be a direct binding partner for LL-37 in monocytes [52], however the intracellular effects mediated by LL-37 and the mechanisms involved remain to be fully understood [2]. LL-37 has the ability to activate multiple receptors and pathways and further investigation within cervical epithelial cells could elucidate if alternative receptors are involved in mediating the effects of IL-8 within these cells.

In conclusion, we have shown that cells of the lower reproductive tract express hCAP18/LL-37 and that expression is increased in cervicovaginal secretions from women with bacterial vaginosis. Moreover in epithelial cells derived from the cervix LL-37 treatment induces IL-8 expression. hCAP18/LL-37 may play an important role in modulating the immune response to invading infection in the lower reproductive tract. Further investigation of these responses may increase the understanding of the physiology and pathophysiology of labour, and lead to strategies for the prevention of infection associated preterm birth.

## Supporting Information

Figure S1 Dose response of LL-37 on IL-8 and IL-6 expression. END E6/E7 cells treated for 6 hours with 0 (control), 5, 10, 25 and 50 µg/ml LL-37. (**A**) IL-8 secretion (n = 3), (**B**) IL-6 secretion (n = 3). Data presented as mean concentration ± SEM (error bars). * p<0.05 (Kruskal Wallace test with Dunn's post-test).
(TIFF)

Figure S2 Time course of LL-37 on IL-8 and IL-6 expression. End E6/E7 cells cultured with 10 ng/ml IL-1β, 1 µg/ml LPS or 25 µg/ml LL-37 with untreated controls over a time course of 0, 2, 4, 6 or 24 hours. (A) IL-8 secretion (n = 3), (B) IL-6 secretion (n = 3). Data presented as mean concentration ± SEM (error bars). *, **, **, p<0.05, 0.01, 0.001 respectively compared with control. (2way ANOVA with multiple comparisons test).
(TIFF)

Figure S3 Effects of LL-37 on IL-8 expression. END E6/E7 and ECT E6/E7 cells treated with scrambled LL-37 (25 µg/ml), D LL-37 (25 µg/ml) and LL-37 (25 µg/ml) with untreated controls. (**A**) END E6/E7 cells (n = 5–14), (**B**) ECT E6/E7 cells (n = 5–14). Data presented as means ± SEM (error bars). ***, p<0.001 compared with untreated control. (One-way ANOVA with Dunnet's post test).
(TIFF)
