# Supplementary figures and images for: Human Cathelicidin Production by the Cervix
Source: PLoS One. 2014 Aug 4;9(8):e103434. doi: 10.1371/journal.pone.0103434 (PMC4121085; doi:10.1371/journal.pone.0103434)

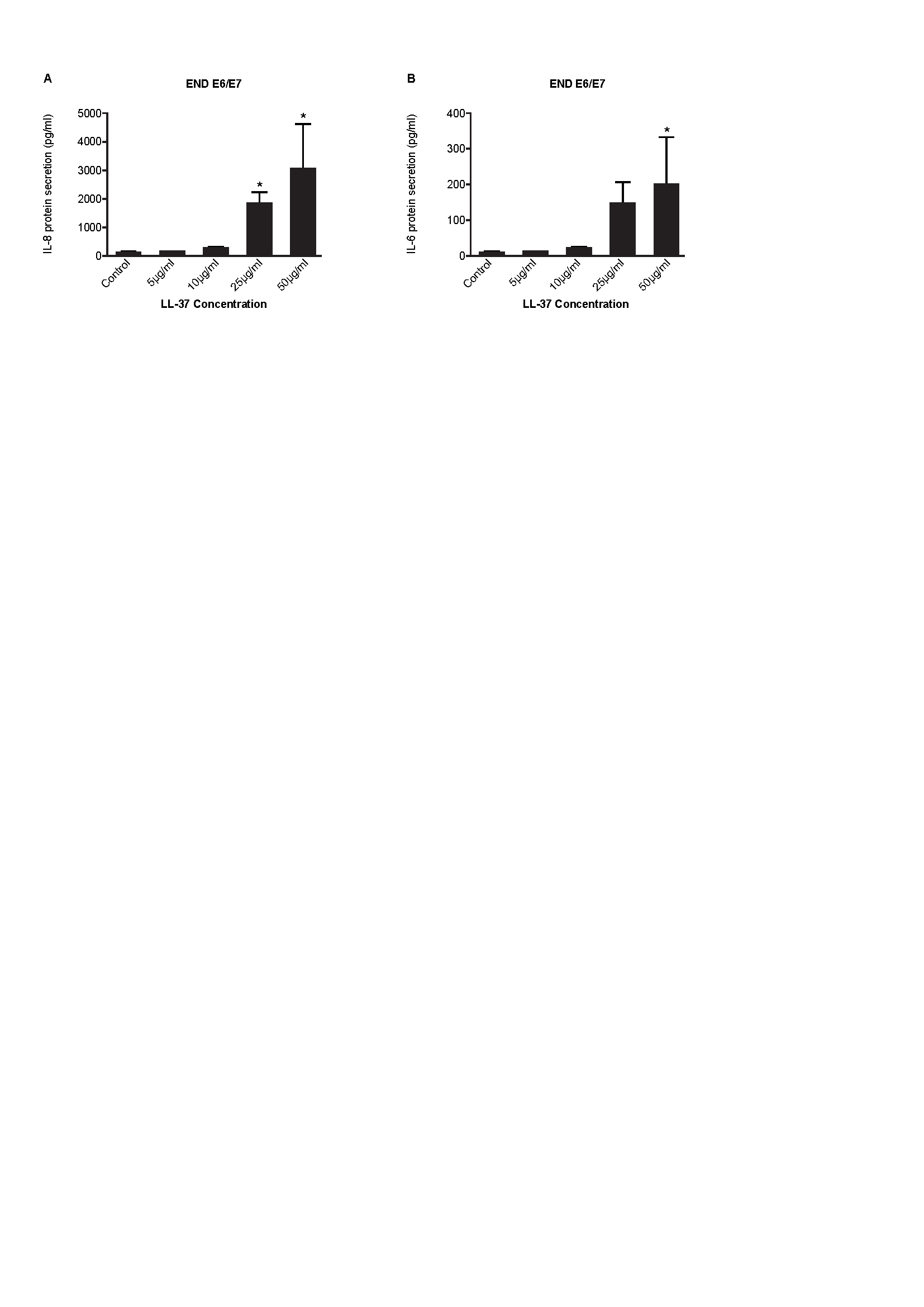

Supplement: Figure S1 — Dose response of LL-37 on IL-8 and IL-6 expression. END E6/E7 cells treated for 6 hours with 0 (control), 5, 10, 25 and 50 µg/ml LL-37. (A) IL-8 secretion (n = 3), (B) IL-6 secretion (n = 3). Data presented as mean concentration ± SEM (error bars). * p<0.05 (Kruskal Wallace test with Dunn's post-test). (TIFF) [file pone.0103434.s001.tif]

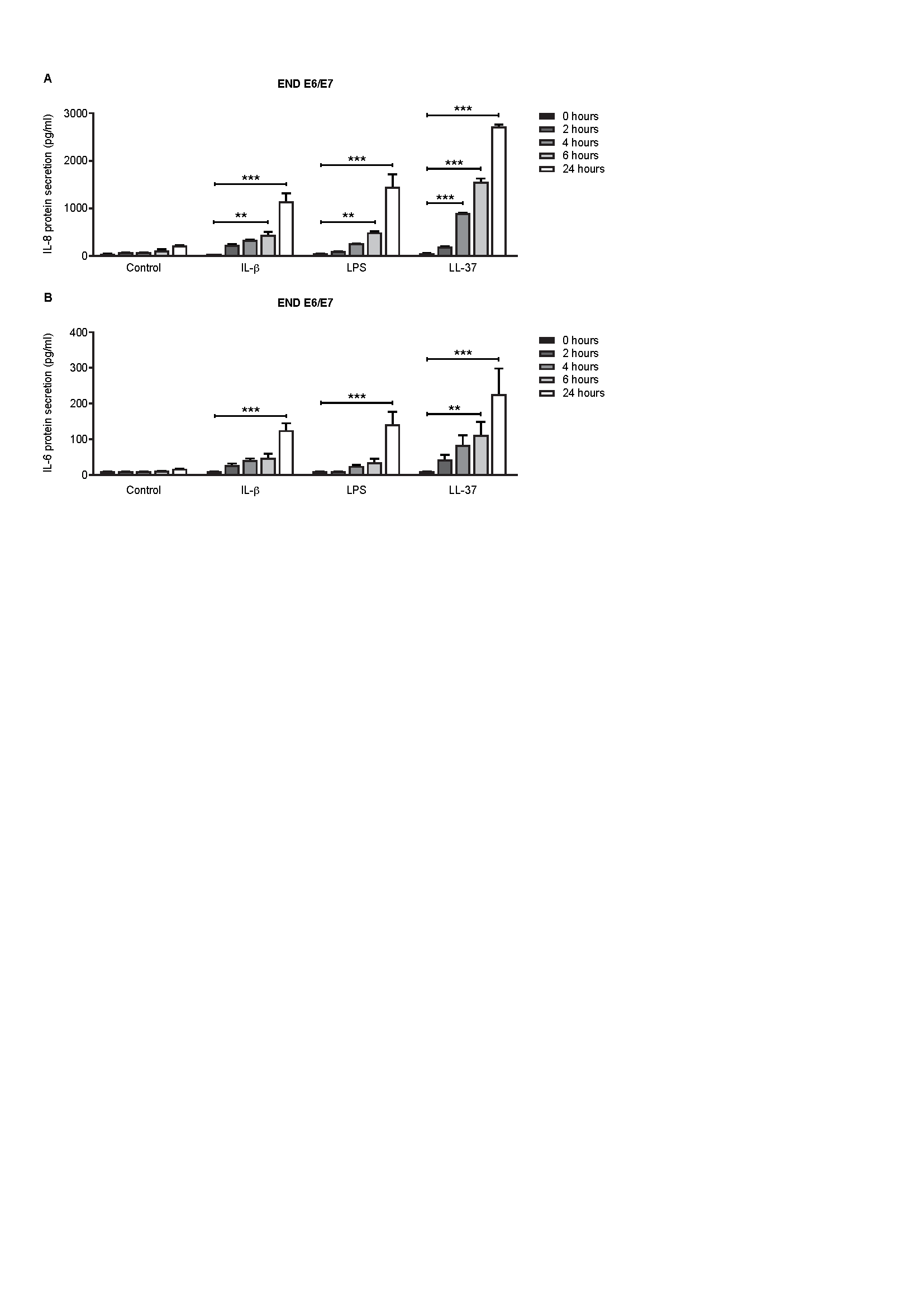

Supplement: Figure S2 — Time course of LL-37 on IL-8 and IL-6 expression. End E6/E7 cells cultured with 10 ng/ml IL-1β, 1 µg/ml LPS or 25 µg/ml LL-37 with untreated controls over a time course of 0, 2, 4, 6 or 24 hours. (A) IL-8 secretion (n = 3), (B) IL-6 secretion (n = 3). Data presented as mean concentration ± SEM (error bars). *, **, **, p<0.05, 0.01, 0.001 respectively compared with control. (2way ANOVA with multiple comparisons test). (TIFF) [file pone.0103434.s002.tif]

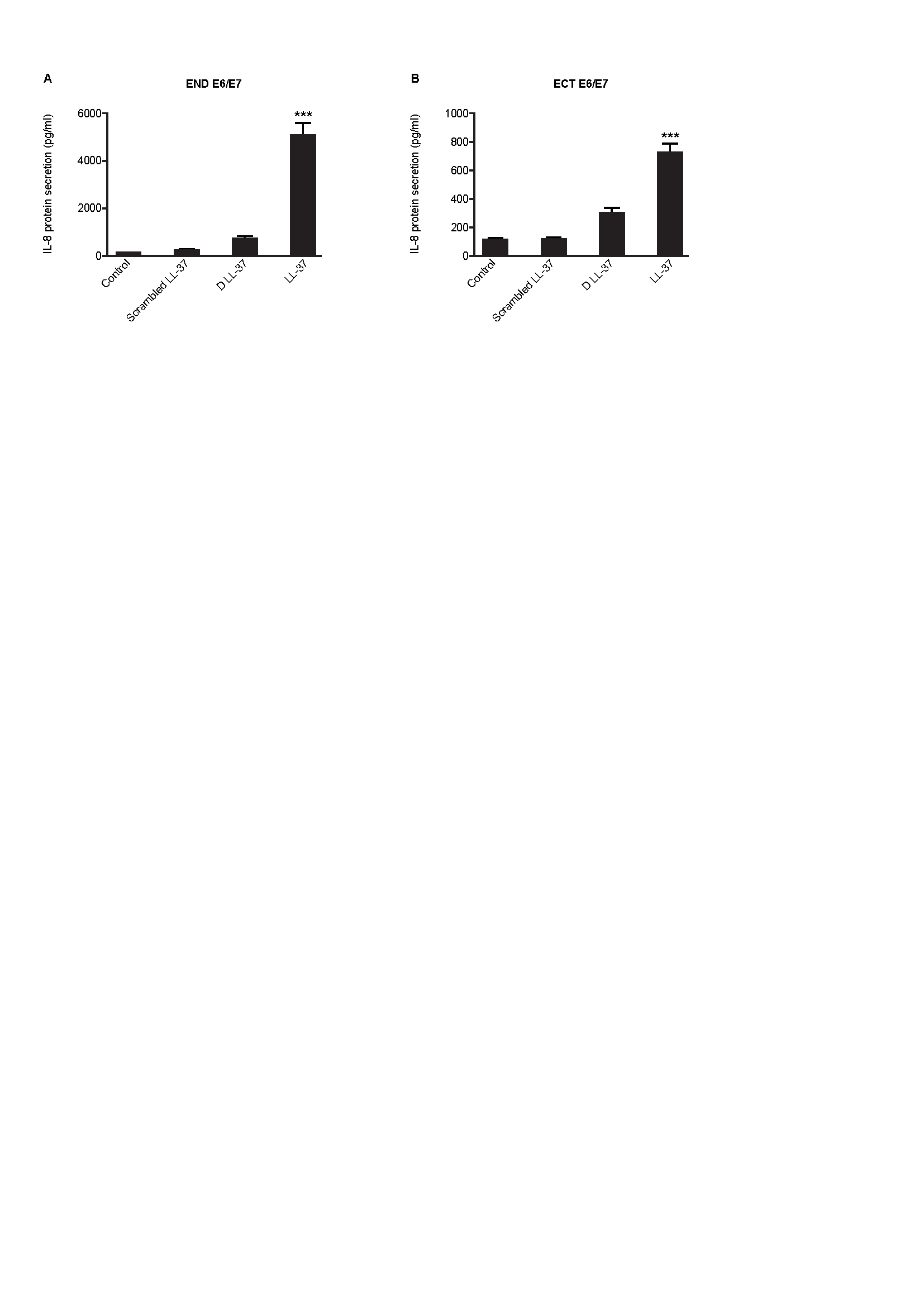

Supplement: Figure S3 — Effects of LL-37 on IL-8 expression. END E6/E7 and ECT E6/E7 cells treated with scrambled LL-37 (25 µg/ml), D LL-37 (25 µg/ml) and LL-37 (25 µg/ml) with untreated controls. (A) END E6/E7 cells (n = 5–14), (B) ECT E6/E7 cells (n = 5–14). Data presented as means ± SEM (error bars). ***, p<0.001 compared with untreated control. (One-way ANOVA with Dunnet's post test). (TIFF) [file pone.0103434.s003.tif]
